# Clinical Efficacy and Safety of Hyperthermic Intraperitoneal Chemotherapy in Colorectal Cancer Patients at High Risk of Peritoneal Carcinomatosis: A Systematic Review and Meta-Analysis

**DOI:** 10.3389/fsurg.2020.590452

**Published:** 2020-11-17

**Authors:** Peng-yue Zhao, Shi-dong Hu, Yu-xuan Li, Ren-qi Yao, Chao Ren, Chang-zheng He, Song-yan Li, Yu-feng Wang, Yong-ming Yao, Xiao-hui Huang, Xiao-hui Du

**Affiliations:** ^1^Department of General Surgery, First Medical Center of Chinese People's Liberarion Army General Hospital, Beijing, China; ^2^Trauma Research Center, Fourth Medical Center of the Chinese People's Liberarion Army General Hospital, Beijing, China; ^3^Department of Burn Surgery, Changhai Hospital, Navy Medical University, Shanghai, China; ^4^Department of Patient Admission Management, First Medical Center of Chinese People's Liberarion Army General Hospital, Beijing, China

**Keywords:** colorectal cancer, HIPEC, peritoneal carcinomatosis, survival, meta- analysis

## Abstract

**Background:** Hyperthermic intraperitoneal chemotherapy (HIPEC) is an effective measure for improving the prognosis of colorectal cancer (CRC) patients with peritoneal carcinomatosis (PC). However, the role of HIPEC in CRC patients at high risk of PC remains controversial. The current systematic review and meta-analysis aimed to evaluate the clinical efficacy and safety of HIPEC in CRC patients at high risk of PC.

**Methods:** We performed a systematic search of PubMed, Embase, Cochrane Library, and other online databases up to July 30, 2020. The clinical data, including overall survival, disease free survival, peritoneal metastasis rate, and postoperative adverse reaction were screened and analyzed after data extraction. Risk ratios (RRs) were applied to analyze these dichotomous outcomes with a random effects model.

**Results:** A total of 6 available clinical studies involving 603 patients were finally included. CRC patients at high risk of PC who proactively underwent HIPEC treatment showed a significantly reduced peritoneal metastasis rate (RR: 0.41, 95% CI: 0.21–0.83, *P* = 0.01; *I*^2^ = 58%) compared to the similarly high-risk in CRC patients who did not receive HIPEC treatment. However, in terms of overall survival (RR: 1.13, 95% CI: 0.97–1.33, *P* = 0.12; *I*^2^ = 77%), disease-free survival (RR: 1.10, 95% CI: 0.75–1.59, *P* = 0.63; *I*^2^ = 53%), progression free survival (RR: 1.85, 95% CI: 0.48–7.14, *P* = 0.37; *I*^2^ = 93%), and postoperative adverse reactions (RR: 0.1.07, 95% CI: 0.36–3.15, *P* = 0.90; *I*^2^ = 78%), there was no significant difference between the HIPEC treatment and control groups.

**Conclusions:** Proactive HIPEC treatment did not show the expected clinical efficacy in prolonging the overall survival time, disease-free survival time, and progression-free survival time of CRC patients at high risk of PC. However, the preemptive administration of HIPEC was associated with a reduced peritoneal metastasis rate and did not cause adverse additional postoperative effects.

## Introduction

Colorectal cancer (CRC) is the most common malignancy of the digestive system, and the latest statistics show that the mortality of CRC ranks second for men and women combined in the United States (1, 2). The peritoneum is the second most likely metastasis site of CRC (3), and the prognosis of CRC patients with peritoneal metastasis (PM) is extremely poor, with a median survival time of 16.8 months (4). Methods of improving the survival rate of CRC patients with PM is the main focus and challenge of CRC research.

In the past few decades, the most common clinical treatment for CRC patients with PM has been systemic intravenous chemotherapy or palliative tumor reduction surgery (5, 6). The emergence of hyperthermic intraperitoneal chemotherapy (HIPEC) treatment greatly alleviated the previous dilemma. HIPEC refers to a novel treatment technique that can prevent and treat primary or secondary peritoneal cancer (PC) by heating the perfusate containing chemotherapeutics to the treatment temperature, and then infusing it into the patients' abdominal cavity for a certain period of time (7, 8). Baratti's study showed that HIPEC was effective in treating CRC patients with PM, and treatment by cytoreductive surgery (CRS) combined with HIPEC extended the median survival time of these patients up to 32 months (9). In addition, multiple clinical studies have shown that patients with peritoneal spread of CRC who undergo CRS plus HIPEC have a 5-year survival rate of 33–58% (10–12). CRS plus HIPEC has gradually become the mainstream treatment for CRC patients with PM on account of its relatively stable safety and efficacy. However, some CRC patients have high risk factors for PM, but their imaging and pathology reports do not confirm peritoneal metastases, such as ovarian metastases from CRC or perforation of the tumor (13). Unfortunately, the detection of PM at an early stage remains elusive due to the lack of typical clinical symptoms and the poor accuracy of imaging (14). In the case of such patients, prophylactic treatment by HIPEC may increase potential clinical benefits. However, some experts have raised different opinions since they believe that the preemptive administration of HIPEC has no clinical basis and may increase adverse reactions in patients (15, 16).

Many systematic reviews and meta-analyses explore the efficacy of HIPEC in CRC patients with PM, but whether HIPEC is effective in CRC patients at high risk of PM is rarely mentioned (17–19). This systematic review and meta-analysis, therefore, aimed to evaluate all published available clinical studies on the efficacy and safety of HIPEC in patients with CRC who are at a high risk of peritoneal carcinomatosis.

## Methods

### Search Strategy

A comprehensive literature search, irrespective of language, was conducted in multiple online databases including PubMed, Embase, and Cochrane Library, up to July 30, 2020. Our strategy that included a combination of exploded medical subject heading (MeSH) terms and the entry terms: “Colorectal Neoplasm,” “Neoplasm, Colorectal,” “Colorectal Carcinoma,” “Carcinoma, Colorectal,” “Colorectal cancer,” “Cancer, Colorectal,” “Colorectal Tumor,” “Tumor, Colorectal,” “Hyperthermic Intraperitoneal Chemotherapy,” “Chemotherapy, Hyperthermic Intraperitoneal,” “Hyperthermic Intraperitoneal Chemotherapies,” “Intraperitoneal Chemotherapy, Hyperthermic,” and “HIPEC.” Studies were also identified by screening the reference lists of systematic reviews on similar subjects. The current study was performed in accordance with the Preferred Reporting Items for Systematic Reviews and Meta-Analysis (PRISMA) statements (20).

### Study Selection

The current meta-analysis included clinical studies comparing the efficacy and safety of HIPEC administration with control groups that did not undergo HIPEC treatment among adult CRC patients at high risk of PC (minimal PC that was completely resected at the same time as the primary tumor; synchronous or metachronous ovarian metastasis; perforated primary tumor inside the peritoneal cavity for some pathologies or iatrogenic reasons), regardless of study type (RCTs or non-RCTs). Considering the limited evidence on gray data, we did not include conference abstract-type research.

The exclusion criteria were as follows: (1) studies with CRC patients who had developed peritoneal metastases or liver metastases; (2) studies with no control groups or with CRC patients in the control group who also underwent HIPEC treatment; (3) studies involving PM that might have originated from areas other than a colorectal origin; (4) ongoing clinical trials; and (5) studies with a lack of sufficient information or without follow-up.

All studies were independently identified by two reviewers. In both the inclusion and exclusion processes, titles and abstracts were initially screened, and any conflicts between two reviewers were resolved by consensus. After screening the titles or abstracts, the full-text was subsequently assessed to determine the eligibility of a particular study.

### Data Extraction and Quality Evaluation

Two reviewers independently extracted data from all eligible studies with a standardized and predesigned form. Characteristics including the first author, year of publication, type of study, the total number of enrolled patients, intervention of treatment and comparison groups, HIPEC methodologies, endpoints, and follow-up times were recorded. Similarly, we resolved inconsistencies in the extracted data by discussion until a consensus was reached.

The risk of bias of each included study was assessed according to its study type. The quality of randomized controlled trials (RCTs) was judged using the Jadad scoring system composed of randomization, double-blinding, withdrawals, and dropouts (21). A score of 0 to 5 was assigned to each trial. If a study scored higher than 3, it was deemed a high-quality study with a low risk of bias. Likewise, the Newcastle Ottawa Scale (NOS) was used to assess the quality of the observational study (cohort study and case-control study) (22). According to this scale, each study was judged through 3 categories (selection, comparability, exposure, or outcomes) and assigned a score of 0 to 9 stars. A study with a score of higher than 5 stars indicated high quality and a low risk of bias.

### Outcome Measurements

We chose 3- or 5-year overall survival (OS) as the primary endpoint due to its generalizability in determining the prognosis of tumor patients. In addition, OS was preferentially reported by the majority of the included studies.

The secondary endpoints included 3- or 5-year disease-free survival (DFS), progression-free survival (PFS) and the incidence of peritoneal metastases or local recurrence. In addition, the rate of postoperative adverse reactions was also analyzed as it reflected the safety of treatment.

### Statistical Analysis

Meta-analysis was conducted using ReviewManager (RevMan 5.3, Copenhagen: the Nordic Cochrane Center, the Cochrane Collaboration, 2014). We applied risk ratios (RRs) for dichotomous outcomes, and pooled proportions were calculated with a 95% confidence interval (95% CI). The *I*^2^ statistic was calculated to evaluate the heterogeneity of each outcome. If *I*^2^ > 50% was considered significant heterogeneity, a random effects model was applied; otherwise, a fixed-effects model was used accordingly. A funnel plot was constructed and visually inspected to assess publication bias. We also conducted Begg's and Egger's tests. A two-sided *P*-value of < 0.05 was deemed statistically significant. Considering the great heterogeneity in risk factors for PC and methodology of HIPEC, we performed subgroup analysis combined with sensitivity analysis to seek potential influencing factors, as well as to validate the consistency and robustness of our findings. All included clinical studies were stratified by type of study (RCT or non-RCT), outcome report, patients subgroup, and HIPEC drug (oxaliplatin alone or not).

## Results

### Literature Search and Characteristics of Included Studies

**Figure 1:**
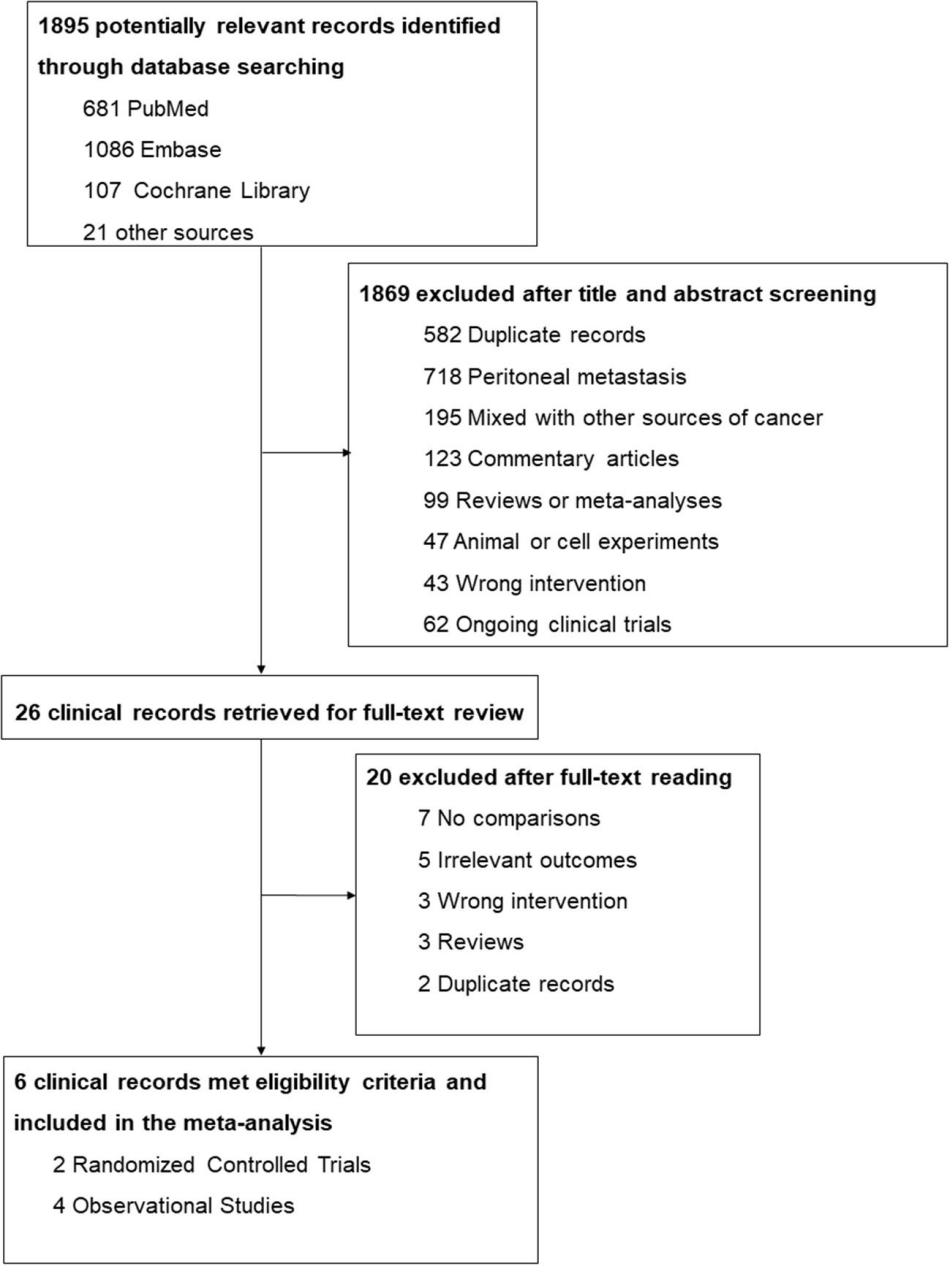
Flowchart for the selection process.

A total of 1,895 potentially relevant records were identified through the database search (681 from PubMed, 1,086 from Embase, and 107 from Cochrane Library) and other sources (6 from conference abstracts and 15 from reference lists). After screening titles and abstracts, 1,869 studies were excluded on account of duplication, already occurring peritoneal metastasis, studies that included other sources of cancer, texts that were not original publications, and those that performed the wrong intervention or comparison. The 26 remaining studies were further evaluated for eligibility via a full-text review, and 20 of them were excluded due to no comparisons, incorrect interventions, and irrelevant outcomes, etc. Finally, 6 clinical studies met the criteria and were included in the meta-analysis. The detailed screening process is shown in Figure 1.

The characteristics of all included studies are presented in Table 1. Six clinical studies (4 observational studies and 2 RCTs) involving 603 patients were enrolled in this systematic review and meta-analysis (23–30). Among all included studies, 2 studies administered HIPEC treatment only in the experimental group, while the remaining 4 studies conducted treatment by HIPEC combined with curative surgery or adjuvant systemic chemotherapy in the intervention group. The majority of studies also chose OS as the primary outcome, while Goéré, Charlotte, and Elias selected 3-year DFS, 18-month PFS, and 3-year DFS, respectively, as their first outcome.

**Table 1:** Characteristic of included clinical studies.

**Study**	**Types of studies**	**No. of patients**	**Intervention**		**Patients subgroup**			**HIPEC methodologies**	**Endpoints**	**Followup time**
			**Treatment group**	**Comparison group**	**Synchronous PC**	**Ovarian mtastases**	**Perforated tumor**			
**Elias et al. 2008** **(****23****)**	Cohort study	13	HIPEC	Complete exploration of the peritoneal cavity	6	1	6	43°C 30 min Oxaliplatin(460 mg/m^2^)	DFS; OS; relapsed in the peritoneum; isolated visceral metastases; postoperative complication	3 years
**Tentes et al. 2011** **(****24****)**	Cohort study	97	HIPEC	Intraperitoneal chemotherapy	Locally advanced colorectal carcinomas			42.5–43°C 90 or 60 min Mitomycin-C (15 mg/m^2^) or Oxaliplatin(130 mg/m^2^)	OS; peritoneal metastases; The incidence of recurrence; postoperative complication	3 years
**Sammartino et al. 2014** **(****25****)**	Case-control study	75	HIPEC + proactive surgical	Standard surgical resection	Advanced colonic cancer			43°C 30 min Oxaliplatin(460 mg/m^2^)	OS; DFS; peritoneal metastases local recurrence; postoperative complication	5 years
**Baratti et al. 2016** **(****26****)**	Cohort study	66	HIPEC + curative surgery+ adjuvant systemic chemotherapy	Standard treatments	18	6	42	42.5°C 60 min Mitomycin-C (3.3 mg/m^2^) and Cisplatin (25 mg/m^2^)	OS; PFS; cumulative PM incidence; postoperative complication	5 years
**Charlotte et al. 2019** **(****27****)**	Randomized controlled trials	202	HIPEC+ Chemotherapy	Chemotherapy	Resectable primary clinical or pathological T4N0–2M0 stage or perforated colon cancer			42°C 30 min Oxaliplatin(460 mg/m^2^)	PFS; OS; peritoneal metastases quality of life; costs	1.5 years
**Goéré et al. 2020** **(****28****)**	Randomized controlled trials	150	HIPEC + systematic second-look surgery	Surveillance	69	20	61	43°C 30 min Oxaliplatin(460 mg/m^2^)	DFS; OS; peritoneal relapse postoperative complications	3 years

*HIPEC, hyperthermic intraperitoneal chemotherapy; DFS, disease-free survival; OS, overall survival; PFS, progression-free survival*.

### Quality Assessment

All the observational studies (3 cohort studies and 1 case-control study) scored higher than 5 stars according to NOS and were thus considered to be of high quality and low risk of bias (Supplementary Tables 1, 2). Only 2 RCTs were included in our study, and these had a score of 3 in the Jadad scoring system (Supplementary Table 3). Both RCTs met the randomization requirements but the blinding method was not effectively implemented, which indicated a high risk of performance bias.

### Primary Outcome: Overall Survival

**Figure 2:**
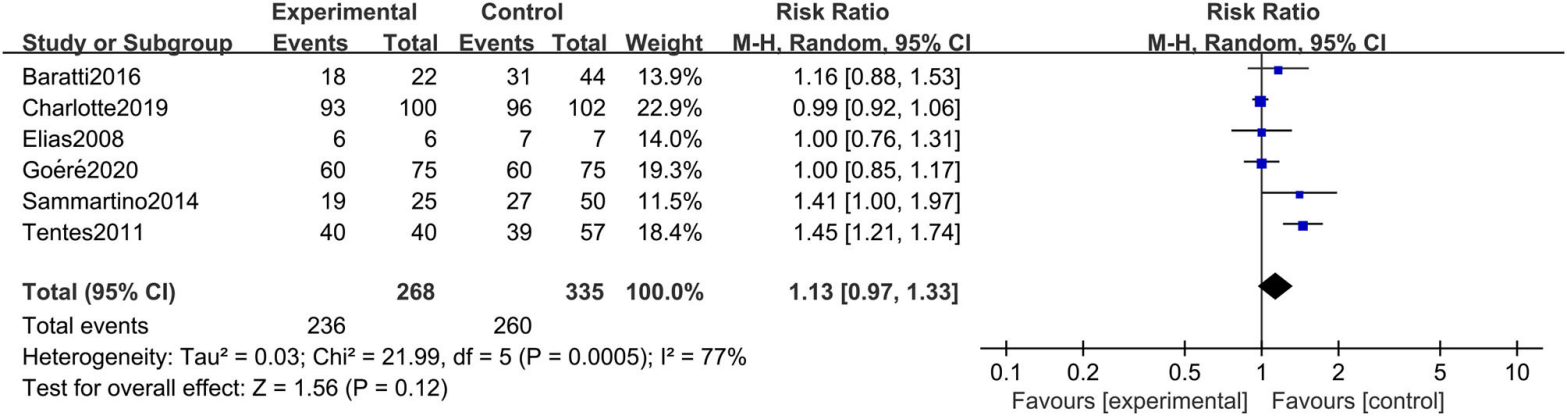
Forest plot of OS comparing the experimental group to control group among CRC patients at high risk of PC.

All six studies included in the systematic review and meta-analysis consistently reported OS as one of the clinical outcomes, while the follow-up time of each study remained irregular (3 years, 5 years, or other timespans). We found that CRC patients at high risk of PC who were undergoing HIPEC treatment had no survival time benefit compared to those undergoing standard treatment without HIPEC (RR: 1.13; 95% CI: 0.97–1.33; *P* = 0.12; *I*^2^ = 77%) (Figure 2). The heterogeneity test showed that the conclusion was proven to be stable by excluding every study, each at a given time.

### Secondary Outcomes

The detailed characteristics of each secondary outcome are summarized in Table 2. As shown in this table, 3 clinical studies reported DFS, and 2 clinical articles mentioned PFS. In addition, 6 and 5 clinical studies selected the incidence of PM and postoperative adverse reactions, respectively, as outcome indicator.

**Table 2:** Summary of primary and secondary outcomes.

**Outcome**	**No. of studies**	**RR**	**95%CI**	***I*^**2**^**	***P*-value**
**Primary endpoint**					
OS	6	1.13	0.97–1.33	77%	0.12
**Secondary endpoint**					
DFS	3	1.10	0.75–1.59	53%	0.63
PFS	2	1.85	0.48–7.14	93%	0.37
PM	6	0.41	0.21–0.83	58%	**0.01**
Postoperative adverse reaction	5	1.07	0.36–3.15	78%	0.90

*OS, overall survival; DFS, disease-free survival; PFS, progression free survival; PM, peritoneal metastasis; RR, risk ratio; CI, confidence interval*.

#### Disease-Free Survival

**Figure 3:**
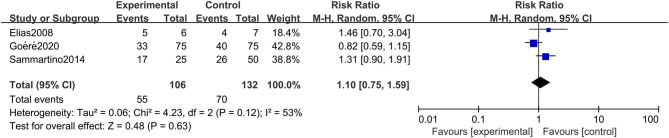
Forest plot of DFS comparing the experimental group to control group among CRC patients at high risk of PC.

A total of 3 studies involving 238 patients reported DFS, and the results showed that HIPEC treatment did not extend the DFS of CRC patients at high risk of PC (RR: 1.10; 95% CI: 0.75–1.59; *P* = 0.63; *I*^2^ = 53%) (Figure 3). Through performing a sensitivity analysis that excluded every study, each at a given time, we found that the conclusion was proven to be stable.

#### Progression Free Survival

**Figure 4:**
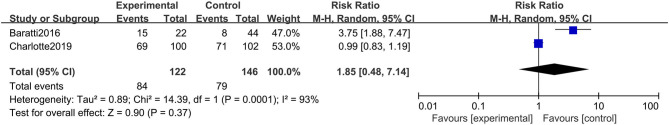
Forest plot of PFS comparing the experimental group to control group among CRC patients at high risk of PC.

Similar to the case of DFS, there were 2 studies with a total of 268 participants choosing PFS as secondary outcomes. Unfortunately, in terms of PFS, the HIPEC group did not show the expected efficacy (RR: 1.85; 95% CI: 0.48–7.14; *P* = 0.37; *I*^2^ = 93%) (Figure 4). However, this outcome displayed fairly high heterogeneity. Through sensitivity analysis, we found that Charlotte's study was the main source of heterogeneity and that if we excluded this study, the robustness of our conclusion would also be affected (RR: 3.75; 95% CI: 1.88–7.47; *P* < 0.01).

#### Incidence of Peritoneal Metastasis

**Figure 5:**
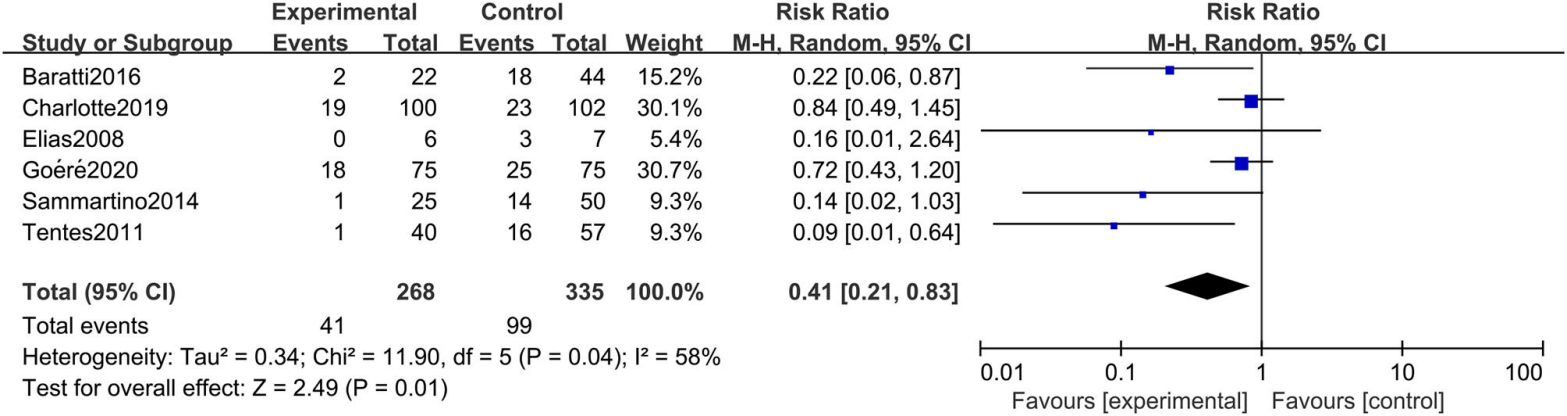
Forest plot of incidence of PM comparing the experimental group to control group among CRC patients at high risk of PC.

The incidence of peritoneal metastasis after treatment was documented in all eligible studies. The results showed that prophylactic HIPEC treatment significantly reduced the incidence of peritoneal metastases in CRC patients at high risk of PC compared to the control group (RR: 0.41; 95% CI: 0.21–0.83; *P* = 0.01; *I*^2^ = 58%) (Figure 5). Although the heterogeneity was relatively high, the conclusion was quite stable after conducting a sensitivity analysis by excluding each study at a time from all qualified clinical studies.

#### Rate of Postoperative Adverse Reactions

**Figure 6:**
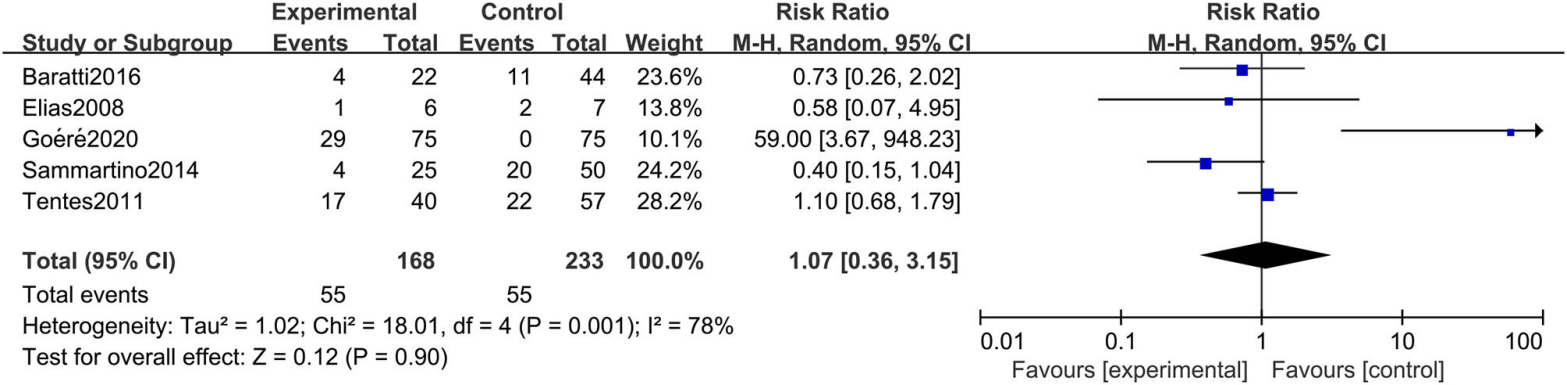
Forest plot of rate of postoperative adverse reactions comparing the experimental group to control group among CRC patients at high risk of PC.

The rate of postoperative adverse reactions was reported in 5 studies. As presented in Figure 6, we observed that there was no significant difference in the rate of postoperative adverse reactions between the two groups (RR: 1.07; 95% CI: 0.36–3.15; *P* = 0.90; *I*^2^ = 78%). Similarly, this conclusion was quite stable after conducting the sensitivity analysis.

### Subgroup Analysis

**Figure 7:**
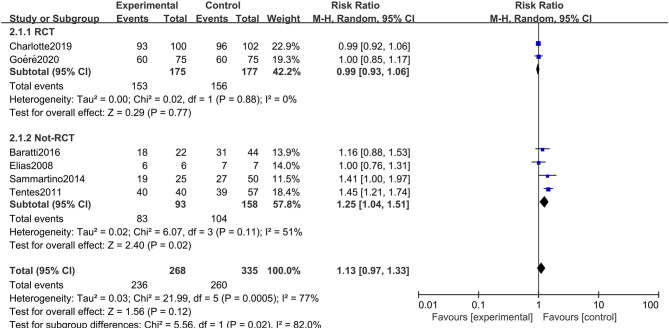
Forest plot of subgroup analysis of RCTs.

**Figure 8:**
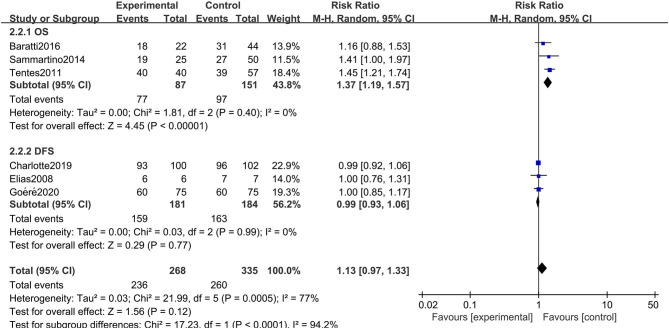
Forest plot of subgroup analysis of studies whose primary outcome was OS.

The rigorous design and standardized implementation of RCTs mean that they have a high level of evidence. Considering that most of the included studies were observational studies, we conducted a subgroup analysis according to the study type. Two RCTs containing 352 patients were enrolled. As presented in Figure 7, although the heterogeneity was significantly reduced, it did not change the conclusion of our research, that is, there was no significant relationship between overall survival and the preventive implementation of HIPEC in the target patients (RR: 0.99; 95% CI: 0.93–1.06; *P* = 0.77; *I*^2^ = 0%). A total of 3 enrolled studies with 238 patients chose OS as the primary endpoint, while the remaining 3 studies chose DFS or PFS as their primary outcome. We observed statistically significant differences in OS between the experimental group and the control group in studies that chose OS as the primary endpoint (RR: 1.37; 95% CI: 1.19–1.57; *P* < 0.01; *I*^2^ = 0%) (Figure 8), which showed that preventive HIPEC treatment could extend the OS of CRC patients at high risk of PC. The reason might be that we unconsciously tended to report positive results when we took OS as the primary outcome.

**Figure 9:**
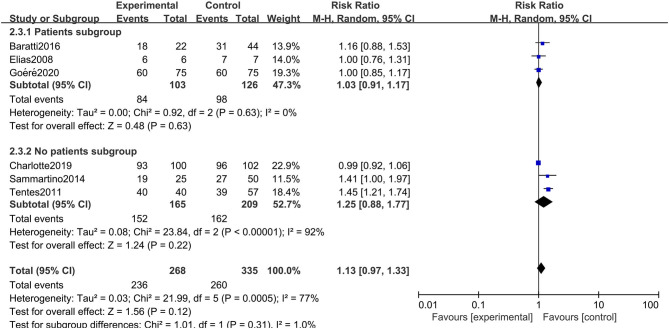
Forest plot of subgroup analysis of patient subgroups.

**Figure 10:**
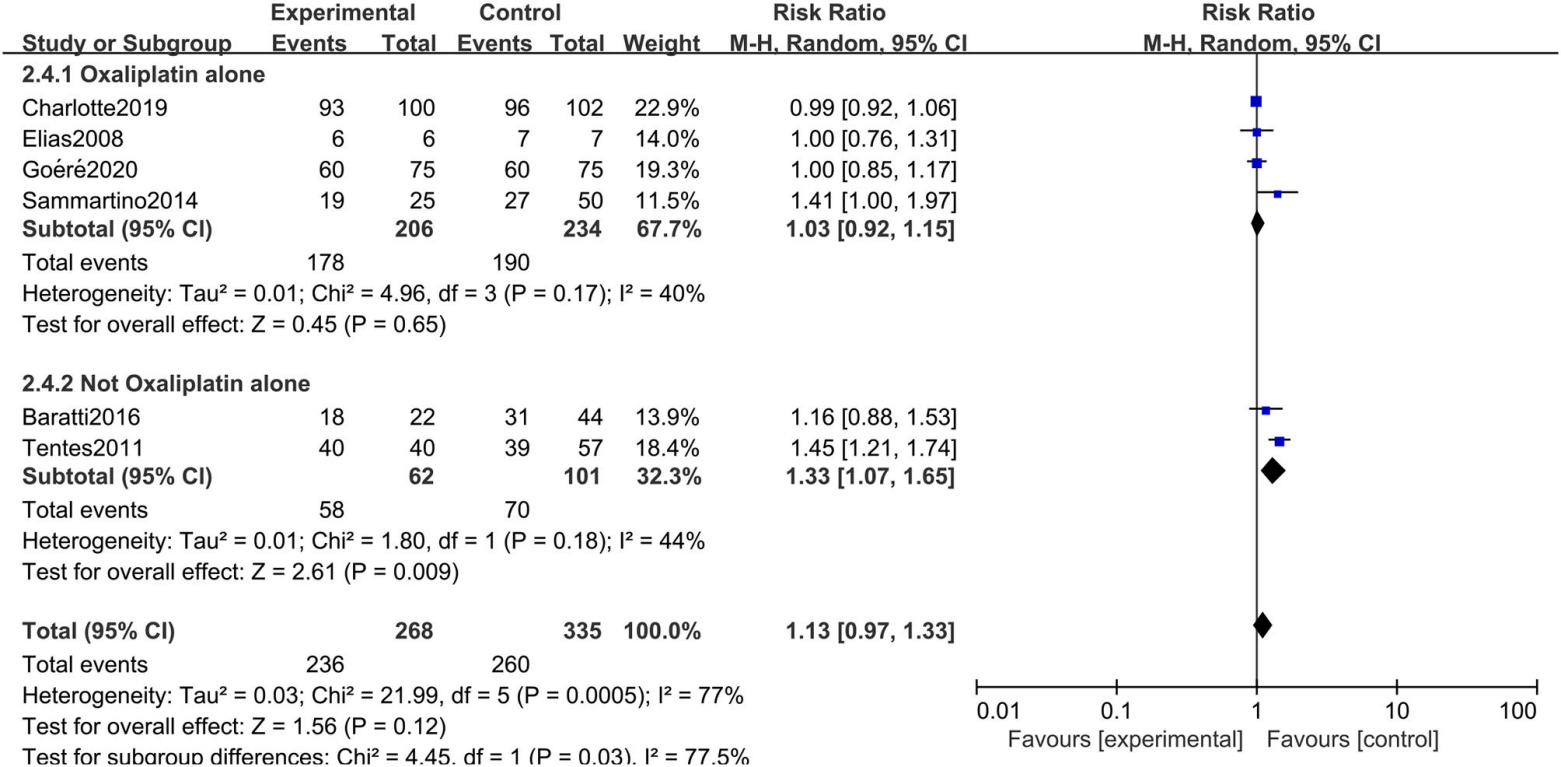
Forest plot of subgroup analysis of HIPEC drugs.

Given the differences in the baseline statistics of the included patients, we also conducted a subgroup analysis according to the patient subgroup. Three studies including 229 patients carefully described and listed the number of colorectal cancer patients with peritoneal metastasis among different high-risk factors. As shown in Figure 9, there were still no significant differences in OS between the two arms when performing a subgroup analysis of studies that carefully described the patients' baseline data (RR: 1.03; 95% CI: 0.91–1.17; *P* = 0.63; *I*^2^ = 0%). Moreover, we conducted a subgroup analysis in the included studies that chose oxaliplatin alone as the chemotherapy drug during HIPEC treatment, and 4 studies involving 440 patients were accordingly enrolled. Surprisingly, choosing oxaliplatin alone as a HIPEC drug did not improve the overall survival time of CRC patients, while when such treatment was combined with other chemotherapy drugs, such as cisplatin or mitomycin-C, CRC patients at high risk of PM had significant survival benefits (RR: 1.33; 95% CI: 1.07–1.65; *P* = 0.009; *I*^2^ = 44%) (Figure 10). The subgroup analysis and sensitivity analyses on primary outcomes are presented in Table 3.

**Table 3:** Subgroup analysis and sensitivity analyses on primary outcomes.

**Subgroup**	**No. of studies**	**No. of patients**	**RR**	**95%CI**	***I*^**2**^**	***P*-value**
**Type of studies**						
RCT	2	352	0.99	0.93–1.06	0%	0.77
Not-RCT	4	251	1.25	1.04–1.51	51%	**0.02**
**Outcome measurement**						
OS	3	238	1.37	1.19–1.57	0%	**<0.001**
DFS or PFS	3	365	0.99	0.93–1.06	0%	0.77
**Patients subgroup**						
Yes	3	229	1.03	0.91–1.17	0%	0.63
No	3	374	1.25	0.88–1.77	92%	0.22
**HIPEC drugs**						
Oxaliplatin alone	4	440	1.03	0.92–1.15	40%	0.65
Not oxaliplatin alone	2	163	1.33	1.07–1.65	44%	**0.009**

*RCT, randomized controlled trials; OS, overall survival; DFS, disease-free survival; HIPEC, hyperthermic intraperitoneal chemotherapy; RR, risk ratio; CI, confidence interval*.

### Publication Bias

**Figure 11:**
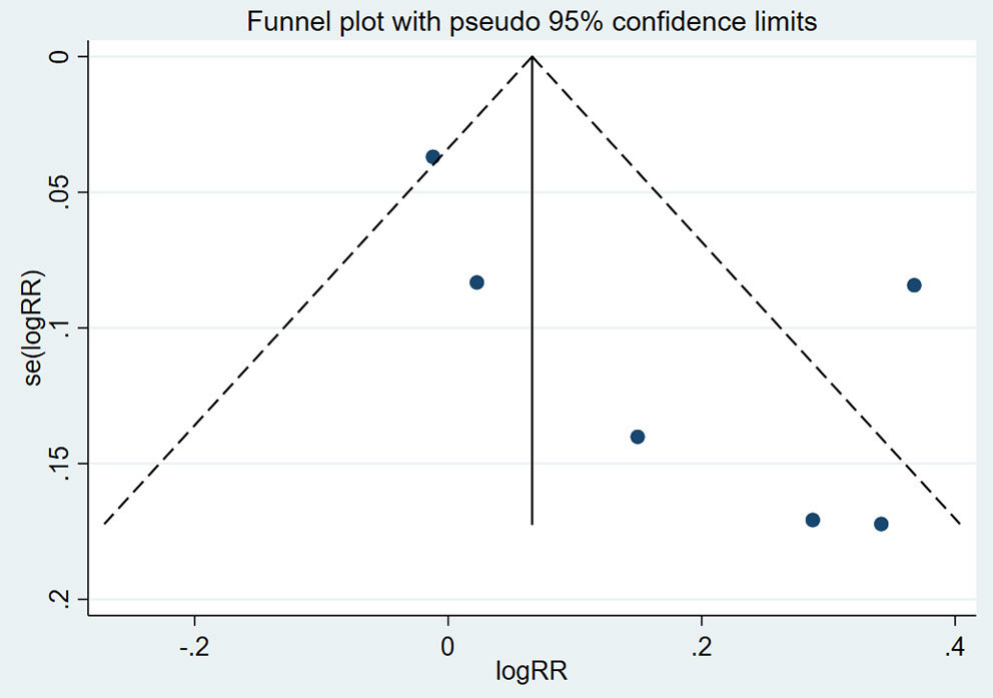
Funnel plot of all included clinical studies.

A funnel plot was constructed to assess the possible publication bias of the primary outcome (Figure 11). There appeared to be no publication bias by visually inspecting the funnel plot. For further verification, we conducted Begg's test and Egger's test to evaluate the funnel plot of OS. The results showed that there was no statistically significant evidence of publication bias (Begg's test: *P* = 0.12; Egger's test: *P* = 0.36).

## Discussion

In this systematic review and meta-analysis, we evaluated the clinical efficacy and safety of the preventative administration of HIPEC among CRC patients at high risk of PC. Unfortunately, we found that preemptive HIPEC did not improve the OS of selected patients with advanced CRC. Additionally, the preemptive HIPEC treatment also showed no benefit in extending DFS or PFS. This may be because HIPEC has clear indications and contraindications. At present, HIPEC is mainly applied to primary or secondary peritoneal tumors in clinical practice. The use of HIPEC therapy in CRC patients at high risk of PM may be a relatively extreme approach, since the existing published RCT research conclusions are similar to ours, and preventive HIPEC does not benefit the long-term survival of the target populations. This conclusion may provide some reference value for other ongoing RCT studies (27, 28). Intriguingly, the incidence of PM was significantly reduced by HIPEC treatment compared to control treatments. In addition, we did not observe an increased rate of postoperative adverse reactions in the experimental group, which verified the safety of preventive HIPEC treatment to some extent. Considering the high heterogeneity, we performed several subgroup analyses according to the primary outcome. The results showed that when we chose all RCTs, in all studies reporting patient subgroups, or those that chose oxaliplatin alone as a HIPEC drug to perform the subgroup analysis, the heterogeneity was markedly reduced while the conclusion was still unchanged. In summary, upfront HIPEC treatment was safe and reduced the incidence of PM, however, there was no significant long-term survival benefit in CRC patients at a high risk of PC, conferred by preventatively administering HIPEC treatment. Therefore, the clinical efficacy of HIPEC in CRC patients at high risk of PC still needs more clinical studies and evidence-based medicine for confirmation.

In the past few decades, there have been several prospective cohort studies and retrospective case-control studies conducted to explore the effectiveness of HIPEC treatment in protecting CRC patients from peritoneal metastasis (15, 31–33). Some related RCTs have also published preliminary results in recent years (27, 29), while to our knowledge, this is the first meta-analysis to summarize previous relevant studies and to evaluate the clinical efficacy and safety of HIPEC in CRC patients at high risk of PC. In 2018, Stamou et al. (14) conducted a similar systematic review that included 12 studies and found that prophylactic HIPEC administered during primary surgery might improve the oncological results (peritoneal recurrence rate, 3-year, and 5-year DFS, and 3-year and 5-year OS) in patients at high risk of developing PC. However, they did not draw firm conclusions due to insufficient evidence. What is exciting about these results, is the fact that the Dutch randomized COLOPEC trial published these clinical results last year. Charlotte et al. (27) finally included 202 patients with clinical or pathological T4N0-2M0-stage tumors or perforated colon cancer and randomly assigned them to a HIPEC treatment group or a control group. Unfortunately, after 18 months of regular follow-up, they found that there was no difference in peritoneal-free survival at 18 months between the two groups (80.9% for the experimental group vs. 76.2% for the control group) and concluded that the administration of adjuvant HIPEC was not advocated on the basis of their trial. Similarly, the randomized phase 3 PROPHYLOCHIP trial also did not show the benefits of a second-look surgery plus HIPEC in patients at high risk of developing colorectal peritoneal metastases (29). Although these two RCTs were conducted based on different protocols, their results questioned the efficacy of preventative HIPEC treatment in CRC patients at high risk of PC. Intriguingly, several prospective cohort studies and retrospective case-control studies reported promising results of preventative HIPEC. Baratti et al. (26, 30), Sammartino et al. (25), Tentes et al. (24), and Elias et al. (23) found that adjuvant HIPEC appeared to improve survival and decrease the incidence of recurrence in advanced colorectal cancer patients who were considered at high risk for peritoneal spread. These were all observational studies with small sample sizes, whose level of evidence was limited to some extent. The diametrically inverse conclusion of RCTs vs. observational studies may be attributed to publication bias, as researchers and publishers tended to report positive results. To reduce the influence of differences in study type as much as possible, we performed a corresponding subgroup analysis, whose results were similar to those of previous research. The subgroup analysis of RCTs did not confirm the benefits of preventive HIPEC in improving the long-term survival of CRC patients at high risk of PC, while the subgroup analysis of observational studies found that prophylactic administration of HIPEC significantly extended the OS, DFS, and PFS of eligible patients. The consistency between the conclusions of our meta-analysis and the results of the RCTs might be due to the larger number of patients included in the RCTs, which meant more weight in the results.

Given the high mortality in CRC patients with peritoneal metastases, early diagnosis and treatment may be the most effective measures to improve their prognosis. Unfortunately, identifying these high-risk patients at an early stage is beyond the sensitivity of current clinical, biological, and imaging techniques. The emergence of HIPEC therapy provides insights into these high-risk patients and the goal of the treatment of CRC with PM has changed from being purely palliative or supportive to being considered curative in selected high risk patients. Unlike traditional surgical treatments and systemic chemotherapy, HIPEC can be used to treat small lesions that are beyond the scope of visual observation. We found that the preemptive administration of HIPEC significantly reduced the incidence of PM. This finding was understandable since preventive HIPEC was a local treatment that could cover all potential peritoneal metastases; consequently, preventive HIPEC can achieve better locoregional control thus reducing local recurrence and peritoneal spread. Moreover, we found that performing HIPEC did not cause additional postoperative adverse effects, the reason might be that HIPEC did not involve an additional surgical intervention and was able to concentrate chemotherapeutic drugs in the abdominal cavity, which was not limited by the contraindications of neoadjuvant chemotherapy to some extent. Unexpectedly, preventative HIPEC treatment did not show the expected superiority in terms of improving OS, DFS, and PFS in CRC patients at high risk of PC. The reasons might be as follows: first, given the limited means of examination, a substantial proportion of the included patients might have developed peritoneal metastasis, for which there was no window of time to administer a preventive intervention. Second, some patients received neoadjuvant systematic chemotherapy before receiving HIPEC treatment. If the drugs used by HIPEC were the same as those in the intravenous chemotherapy, the efficacy of HIPEC might be affected because this neoadjuvant treatment potentially induced a certain degree of resistance to certain drugs in the tumor cells; third, the adjuvant HIPEC procedure commonly used in the literature involved adding chemotherapy drugs to the infusion solution for 30 min at a minimum infusion temperature of 42°C, and a single 30-min exposure of malignant cells to chemotherapeutic drugs might also be too short to obtain a clinically relevant antitumor effect; additionally, the optimal timing of early surgery and HIPEC treatment remained unclear and requires further evaluation. If the treatment time was not appropriate, the clinical efficacy would also be affected. Numerous related to ongoing clinical trials have not yet reported their results, including the PROMENADE (NCT02974556) trial, HIPECT4 (NCT02614534) trial, CHECK (NCT03914820) study, and some other similar trials worldwide (34–39). The outcomes of these clinical trials might contribute to drawing a more definitive conclusion on the efficacy and safety of preventative HIPEC treatment in CRC patients at high risk of PC.

Several limitations should be taken into account in this systematic review and meta-analysis. First, we enrolled only 6 clinical studies including 2 RCTs and 4 observational studies with small sample sizes, so it was difficult to confirm the conclusion. Moreover, RCTs and observational studies are fundamentally different. Mixing them for a meta-analysis may lead to unconvincing results. Considering this limitation, we conducted strict quality assessments on all included studies. The evaluation results showed that all included RCTs and observational studies were of high quality and low risk of bias. We ruled out the existence of publication bias through Begg's test and Egger's test. Second, the heterogeneity of some outcomes was relatively high, indicating a large variability in results among studies. We further performed a subgroup analysis combined with sensitivity analysis to find potential influencing factors. Third, the methodologies of HIPEC, such as the timing, techniques, duration, and agents, were disparate across the different enrolled studies. Moreover, the treatments in the control groups including surveillance, systematic chemotherapy, and standard surgical resection, were also uneven. All of the above factors might have affected the robustness of our conclusions. Finally, we could not evaluate the quality of evidence of outcomes in line with the Grading of Recommendations, Assessment, Development, and Evaluation (GRADE) criteria due to the inconsistent types of eligible studies.

## Conclusion

The current systematic review and meta-analysis did not show the expected superiority of preventative HIPEC treatment in improving OS, DFS, and PFS in CRC patients at high risk of PC. However, the preemptive administration of HIPEC was found to significantly reduce the incidence of PM and, at the same time, did not cause additional postoperative adverse effects.

## Data Availability Statement

All datasets generated for this study are included in the article/Supplementary Material.

## Author Contributions

P-yZ, S-dH, and Y-xL conceived the study and co-wrote the paper. P-yZ and S-dH extracted all data. Y-xL, R-qY, CR, C-zH, S-yL, and Y-mY undertook and refined the search. P-yZ, R-qY, and CR undertook statistical analyses. Y-fW, Y-mY, X-hH, and X-hD helped to revise the intellectual content. All authors read and approved the final manuscript.

## Conflict of Interest

The authors declare that the research was conducted in the absence of any commercial or financial relationships that could be construed as a potential conflict of interest.

## Abbreviations

CRC: Colorectal cancer
PC: peritoneal carcinomatosis
PM: peritoneal metastasis
HIPEC: hyperthermic intraperitoneal chemotherapy
CRS: cytoreductive surgery
RRs: Risk Ratios
95%CI: 95% confidence intervals
OS: overall survival
DFS: disease-free survival
PFS: progression-free survival
RCTs: randomized controlled trials.
