# Supplementary material for: Clinical Efficacy and Safety of Hyperthermic Intraperitoneal Chemotherapy in Colorectal Cancer Patients at High Risk of Peritoneal Carcinomatosis: A Systematic Review and Meta-Analysis
Source: Front Surg. 2020 Nov 17;7:590452. doi: 10.3389/fsurg.2020.590452 (PMC7705102; doi:10.3389/fsurg.2020.590452)
Supplement: Supplementary file 1 [file Table_1.DOCX]

**Supplemental Table S1. The Newcastle-Ottawa Scale (NOS) of case control studies or cohort study**

| **Study** | **Types of**  **studies** | **Selection**  **🟑 🟑 🟑 🟑** | **Comparability**  **🟑 🟑** | **Exposure/Outcomes**  **🟑 🟑 🟑** | **Total**  **score** | **Risk of**  **bias** |
| --- | --- | --- | --- | --- | --- | --- |
| **Baratti et al,**  **2016** | CS | **🟑 🟑 🟑** | **🟑 🟑** | **🟑 🟑** | 7 | Low risk |
| **Sammartino et al,**  **2014** | CCS | **🟑 🟑 🟑** | **🟑 🟑** | **🟑 🟑** | 7 | Low risk |
| **Tentes et al,**  **2011** | CS | **🟑 🟑 🟑** | **🟑 🟑** | **🟑 🟑** | 7 | Low risk |
| **Elias et al,**  **2008** | CS | **🟑 🟑** | **🟑 🟑** | **🟑 🟑** | 6 | Low risk |

Abbreviations: CS, Cohort study; CCS, case-control study.
