# Supplementary material for: Clinical Efficacy and Safety of Hyperthermic Intraperitoneal Chemotherapy in Colorectal Cancer Patients at High Risk of Peritoneal Carcinomatosis: A Systematic Review and Meta-Analysis
Source: Front Surg. 2020 Nov 17;7:590452. doi: 10.3389/fsurg.2020.590452 (PMC7705102; doi:10.3389/fsurg.2020.590452)
Supplement: Supplementary file 3 [file Table_3.DOCX]

**Supplemental Table S3. Jadad scale of randomized controlled trials included in the meta-analysis.**

| **Study** | **Randomization** | **The method of randomization was described and appropriate** | **The study was described as randomized** | **Double- blinding** | **The method of double blinding was described and appropriate** | **The study was described as double blind** | **Withdrawals and dropouts** | **Total Jadad score** |
| --- | --- | --- | --- | --- | --- | --- | --- | --- |
|  |  |  |  |  |  |  |  |  |
|  |  |  |  |  |  |  |  |  |
|  |  |  |  |  |  |  |  |  |
| Goéréet al | Yes | Yes | No | No | No | No | Yes | 3 |
| Charlotte et al | Yes | Yes | No | No | No | No | Yes | 3 |
